# SG-APSIC1110: Project quality development of zero stock

**DOI:** 10.1017/ash.2023.104

**Published:** 2023-03-16

**Authors:** Tussanee Nimnaparoj

**Affiliations:** Central Sterilizing Services Association, Bankok, Thailand

## Abstract

**Objectives:** Many medical devices and equipment have been reserved in hospital wards and outpatient departments. Among these items, >80% are not often used but are being reserved for emergency situations. We aimed to reduce the number of unnecessarily reserved medical devices and to reduce the cost of unnecessary resterilization of devices and equipment. **Methods:** The central sterile supply department (CSSD), in coordination with other 13 wards within the Thammasat University Hospital, established a standard action plan for improving the efficiency of medical supply stocking and storage. Medical equipment and/or devices were returned to the CSSD, which acted as the center of management and distribution. The CSSD also tracked and solved problems that occurred and reevaluated practice guidelines. User satisfaction was evaluated and statistic data were collected and analyzed. **Results:** Wards no longer reserve medical equipment. Thus, no repeated sterilization was needed for unused medical equipment from the participated 13 wards, and sufficient medical equipment was available for various wards when needed. This project helped reduce the cost of purchasing medical equipment, especially for a newly opened ward. The storage of all medical equipment and devices complied with the practice guidelines because the CSSD storage room had a standard temperature and humidity control system. **Conclusions:** In this project, the CSSD cooperated with the 13 participating wards. As the result of centralizing the supplies, the CSSD has sufficient medical equipment and devices for all other wards, including a newly opened ward. The hospital benefitted from reduced costs of purchasing new medical equipment for a newly opened ward as well as the cost savings of eliminating unnecessary resterilization of unused devices and equipment.

